# Managing biodiversity in the Anthropocene: discussing the Nature Futures Framework as a tool for adaptive decision-making for nature under climate change

**DOI:** 10.1007/s11625-022-01200-4

**Published:** 2022-09-21

**Authors:** Juliano Palacios-Abrantes, Renuka Badhe, Amanda Bamford, William W. L. Cheung, Wendy Foden, Catarina Frazão Santos, Kerry-Anne Grey, Nicola Kühn, Kristi Maciejewski, Henry McGhie, Guy F. Midgley, Izak P. J. Smit, Laura M. Pereira

**Affiliations:** 1 Center for Limnology, University of Wisconsin-Madison, Madison, USA; 2 Changing Oceans Research Unit, The Institute for the Oceans and Fisheries, University of British Columbia, Vancouver, Canada; 3 European Polar Board, The Hague, The Netherlands; 4 Division of Evolution and Genomic Sciences, Faculty of Biology, Medicine and Health, School of Biological Sciences, University of Manchester, Manchester, UK; 5 Global Change Biology Group, Department of Botany and Zoology, Stellenbosch University, Matieland, South Africa; 6 South African National Parks, Cape Research Centre, Tokai Park, Cape Town, South Africa; 7 Climate Change Specialist Group, Species Survival Commission, International Union for Conservation of Nature, Gland, Switzerland; 8 MARE–Marine and Environmental Sciences Center / ARNET–Aquatic Research Network, Department of Animal Biology, Faculty of Sciences, University of Lisbon, Lisbon, Portugal; 9 Environmental Economics Knowledge Center, Nova School of Business and Economics, New University of Lisbon, Lisbon, Portugal; 10 School of Geography and the Environment, University of Oxford, Oxford, UK; 11 Centre for Sustainability Transitions, School of Public Leadership, Stellenbosch University, Stellenbosch, South Africa; 12 Curating Tomorrow, 40 Acuba Road, Liverpool, UK; 13 Scientific Services, South African National Parks, Skukuza, South Africa; 14 Sustainability Research Unit, Nelson Mandela University, George, South Africa; 15 Copernicus Institute for Sustainable Development, Utrecht University, Utrecht, The Netherlands; 16 Stockholm Resilience Center, Stockholm, Sweden; 17 Global Change Institute, University of the Witwatersrand, Johannesburg, South Africa

**Keywords:** Nature Futures Framework, Social-ecological systems, Nature’s contributions to people, Climate change, Nature values

## Abstract

Conservation approaches to social-ecological systems have largely been informed by a framing of preserving nature for its instrumental societal benefits, often ignoring the complex relationship of humans and nature and how climate change might impact these. The Nature Futures Framework (NFF) was developed by the Task Force on scenarios and models of the Intergovernmental Science-Policy Platform on Biodiversity and Ecosystem Services as a heuristic approach that appreciates the diverse positive values of nature and its contribution to people. In this overview, we convene a group of experts to discuss the NFF as a tool to inform management in social-ecological systems facing climate change. We focus on three illustrative case studies from the global south across a range of climate change impacts at different ecological levels. We find that the NFF can facilitate the identification of trade-offs between alternative climate adaptation pathways based on different perspectives on the values of nature they emphasize. However, we also identify challenges in adopting the NFF, including how outputs can be translated into modeling frameworks. We conclude that using the NFF to unpack diverse management options under climate change is useful, but that there are still gaps where more work needs to be done to make it fully operational. A key conclusion is that a range of multiple perspectives of people’s values on nature could result in adaptive decision-making and policy that is resilient in responding to climate change impacts in social-ecological systems.

## Introduction

Climate change presents one of the greatest threats to biodiversity and society (IPCC 2019; Bellard et al. 2012; Urban 2015; Pecl et al. 2017). Over the last century, global average air temperature has increased over 1 °C, rainfall patterns have changed in intensity and frequency resulting in heavier droughts and precipitation events (IPCC 2019). Impacts are also evident in the ocean, which has absorbed more than 90% of the excess heat and about 20–30% of the extra carbon dioxide produced by climate change over the past century. This has resulted in increasing sea surface temperature by 0.63 °C (± 0.6 °C) since the pre-industrial era and the acidification of open ocean waters by 0.017–0.027 pH units per decade since the late 1980s (IPCC 2019). Such changes have impacted multiple ecological processes at different biological levels from organisms (e.g., genetic diversity), to populations (e.g., migration patterns), to communities (e.g., species interactions) in both oceanic (Scheffers et al. 2016; Pecl et al. 2017) and land systems (Tilman et al. 2017).

As ecosystems are affected by a changing climate, all life on earth needs to adapt to a new reality (IPCC 2014a, b). Doing this effectively relies on developing and constantly refining models that consider current and past knowledge on the complexity of social-ecological systems while acknowledging the possible pathways (scenarios) that society could take in response to climate change (IPCC 2014a, b). The Fifth Assessment report of the Intergovernmental Panel on Climate Change (IPCC) introduced the idea of ‘climate-resilient development pathways’ (CRDPs) as key responses to the threat of climate change (IPCC 2014a, b). CRDPs are not merely scenarios to envision possible futures, but processes that can be used in the form of deliberation and implementation to address societal values and their inevitable trade-offs (O'Brien 2016; Harris et al. 2017). They provide a mechanism through which to situate transformation, resilience, equity and well-being into decision-making processes across various scales, while acknowledging the complex reality of specific places and communities (Harris et al. 2017; Fazey et al. 2018; Gajjar et al. 2018; Klinsky and Winkler 2018). However, to date, the concept of CRDPs has mainly been applied to challenges that focus on societal adaptation needs and have tended to neglect the ecosystems within which humans are embedded (IPCC 2018).

Most of the knowledge generated for biodiversity conservation and management of ecosystems has largely been informed by a framing of preserving nature for its societal benefits (Wyborn et al. 2021). This is despite a growing recognition that human communities have diverse values for nature, often deeply embedded in cultural relations (Rocha and Liberato 2015; Pascual et al. 2017; Green et al. 2015; Mace 2014). However, these values are rarely accounted for in adaptation responses to climate change (Adger et al. 2013). One reason for this is the lack of a framework that is able to recognize multiple (desired) futures for nature and society, and which acknowledges potential trade-offs between values when incorporating aspects like climate change into models (Pereira et al. 2020; Lindquist et al*.* 2017; Rosa et al. 2017; IPBES 2016; Díaz et al. 2015).

The recent development of the Nature Futures Framework (NFF) by the Task Force on Scenarios and Models as part of the ongoing work conducted by the Intergovernmental Science-Policy Platform on Biodiversity and Ecosystem Services (IPBES) is a promising tool to identify futures for nature and society in the conservation space (Pereira et al. 2020). The NFF builds on an ongoing scholarship that engages with the need for a diversified framing on values of nature and its contributions to people (NCP), including an emphasis on relational values (Chan et al. 2012). It aligns with the historical conservation approaches proposed by Mace (2014), namely “Nature for itself” and “Nature despite people”, “Nature for people”, and “People and Nature”. Its core contribution is that it offers a single framework to acknowledge these diverse positive values for human–nature relationships grouped into three main areas: nature for nature (NN), nature for society (NS), and nature as culture (NC). The framework specifically intends to be a tool for identifying and creating indicators for the computer modeling and scenario designing community allowing plurality of perspectives to be held in different times, contexts and spaces (Kim et al. 2021; Pereira et al. 2020; IPBES 2019).

The three NFF perspectives form a continuum that is represented in a triangle, which can be employed in research activities across different scales and sectors. The vertices of the triangle serve as reference points for analyzing differences and convergences in the three extreme value positions. However, in practice, a community discussing a management action (e.g., spatial zonification) could have some members valuing more a certain area of the triangle (e.g., an NN value perspective) while other members value another area (e.g., an instrumental NS value) (Pereira et al. 2020). Thus, the emphasis on the corners of the triangle is not intended to represent the reality of the world, but rather to open up the full extent of value perspectives by including those with an extreme focus on one type of value perspective at the corners of the triangle. See Pereira et al. (2020) for a more in-depth discussion on how the NFF is situated relative to other frameworks, and the longer term research strategy that seeks to apply it to multiple case studies.

The NFF contributes to filling two main gaps in the use of scenarios and models to inform decision-making in conservation of biodiversity and ecosystem services: (1) to recognize the plurality of possible value perspectives placed on nature, and (2) to incorporate such diverse value perspectives into scenarios and models (IPBES 2019). However, to do this, we need to understand what the boundaries of the triangle (i.e., what is considered as desirable versus not neccessary in the first place). The NFF needs to be used in diverse settings to ensure that it is genuinely actionable (Pereira et al. 2020). The objective of this paper is to identify a potential use of the NFF as a tool for recognizing multiple management options towards more desirable futures for nature under climate change. We propose that the inclusion of more diverse values for nature through the NFF can be a steppingstone for opening up more decision-making pathways under climate change impacts, as suggested by the IPCC’s CRDPs (IPCC 2018) and others (Pascual et al. 2021). Our starting premise is that the NFF can be used as a boundary object for opening plural perspectives, and as such provides a framework for coherently holding a plurality of value perspectives. Consequently, the recognition of a wider range of how people value nature opens alternative management options to account for these different values. A wider range of options means identifying more trade-offs and co-benefits between the options, thereby making the decision-making process more transparent and potentially more resilient to a changing world (IPCC 2018; Pascual et al. 2021).

Here, we focus on three illustrative case studies from the global south across a range of climate change impacts at different ecological levels: (1) *Aloidendron dichotomum* tree in southern Africa, (2) woody encroachment of savannas of southern America and Africa, and (3) the Galapagos Marine Reserve in the Pacific ocean. Through these case studies, we illustrate the potential applications of the NFF to generate a diverse set of pathways in response to a changing climate. We center our discussion on comparing the different case studies under the NFF lens while also identifying trade-offs and co-benefits between and across NFF values. Finally, we discuss ways to incorporate the NFF into computer modeling exercises to support decision-making and close with benefits and challenges for future research and management. This opinion piece is the result of a workshop attended by a diverse group of professionals (i.e., scientists, managers, and practitioners) gathered at a conference session to discuss how the NFF could support ecosystem management under climate change.

## Materials and methods

This paper is the result of a conference session introducing the NFF to the climate change and conservation community at the Species on the Move conference, held in Kruger National Park, South Africa, in July of 2019. Here, a diverse group of 15 professionals, including academics from disciplines as far ranging as climate and ecological modeling, botany, fisheries, and economics, representatives from regional decision-making bodies, museum curators and national park managers, joined the workshop for a discussion on the NFF and whether they thought it could be usefully applied in their work. As a result, it was decided that it would be important to think about the framework across a range of different contexts to see whether it was possible to use as a heuristic for opening up alternative management options. This became the basis for this paper.

Methodologically, we employ a comparative case study approach to open up alternative management options for conservation under the threat of climate change. As such, the findings are meant to be illustrative and not definitive (Knight 2001). In order to ensure that we discussed the NFF across a range of different terrestrial and marine social-ecological systems, while keeping the study manageable, we decided on three specific selection criteria for the case studies: (1) in order to address biases in global conservation information (Di Marco et al. 2017), cases would come from the global South; (2) they needed to represent different ecological levels: on an individual species, on an ecological process, and on a specific ecosystem; and (3) represent real-life challenges of social-ecological systems due to climate change in a context of multiple stakeholders and values (Table 1). The specific case studies were selected based on having a scientist or manager present at the workshop who could contribute their expertise and experience from a specific case study area. For each case study, experts were asked to address a same set of questions as a group based on previous knowledge and data on the matter.

**Table 1:** Summary of case studies, main threat from climate change and related natural response

Case study	Region	Ecological level	Ecosystem	Main threat from climate change	Natural response to threat
1. *Aloidendron dichotomum*	Namibia and South Africa	Species	Desert/Shrubland	Increased air temperature	Shift in species’ distribution
2. Woody encroachment	Southern Africa and America	Community	Savannas	Increased CO_2_ and rainfall changes	Increased woody cover
3. Galapagos Marine Reserve	Galapagos, Ecuador	Ecosystem	Tropical marine	Intensification of El Niño	Biodiversity loss

Overall, experts were required to set each case study under the lens of the NFF and identify how climate change is impacting each component of the NFF through a business as usual (BAU) policy. By BAU, we mean a continuation of current practices and trends. Moreover, such impacts were categorized as positive or negative feedback or trade-offs*,* depending on the relation between the BAU policy and the NFF values. Positive feedback referred to when the BAU policy had a positive impact on the NFF value (e.g., a current policy that fosters biodiversity would have positive feedback on NN). On the contrary, negative feedback was considered when the BAU negatively impacted the NFF value (e.g., using non-selective extractive methods would negatively impact NN). Lastly, we consider a trade-off when the BAU policy had both positive and negative impacts to the NFF value (e.g., from a NC perspective, a policy that promotes mechanical clearing of woody encroachment has positive feedback on landowners by the reduction of costs but negatively impacts workers due to job reduction) or when the policy did not directly respond to climate change. It is worth mentioning that, while we mostly focus in one BAU scenario, future research considering multiple management options not used at the moment could further diversify pathways.

In the following sections, we provide a brief description of each case study where we describe the climate change impacts and identify the current management options, all of this under the lens of the three components of the NFF (see “Case study description”). Later, we use the information from the case studies to analyze what impact climate change could have on different NCP across the three value perspectives in each of the examples if the current management regime were to be effective (see “Discussion”). Finally, the importance of recognizing and opening up plural values, analyzing the potential impacts of climate change on what these perspectives most value and then weighing up management options offers a much more nuanced and resilient suite of management options (see “Discussion”).

## Results

### Case study description

**Fig. 1:**
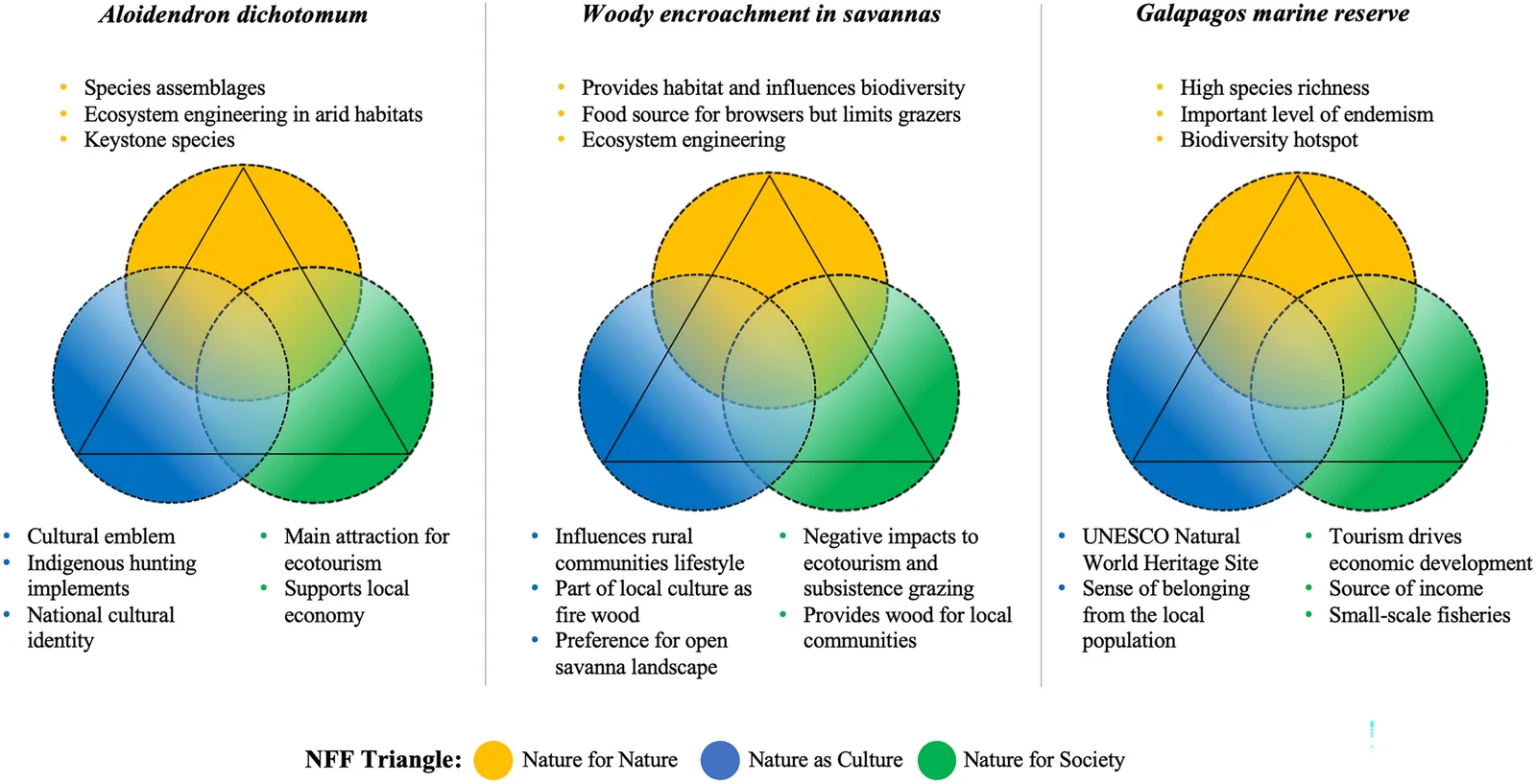
The Nature Futures Framework illustrating the three main value perspectives for each case study (Adapted from Pereira et al. 2020)

In the following section, we briefly describe the three case studies (i.e., *Aloidendron dichotomum*, woody encroachment, and the Galapagos Marine Reserve). Specifically, for each case study, we present the current climate change impacts and describe the current management options. Moreover, we describe the case study under the lens of all three NFF components (nature for nature—NN, nature for society—NS, and nature as culture—NC) (Fig. 1).

### *Aloidendron dichotomum*

**Fig. 2:**
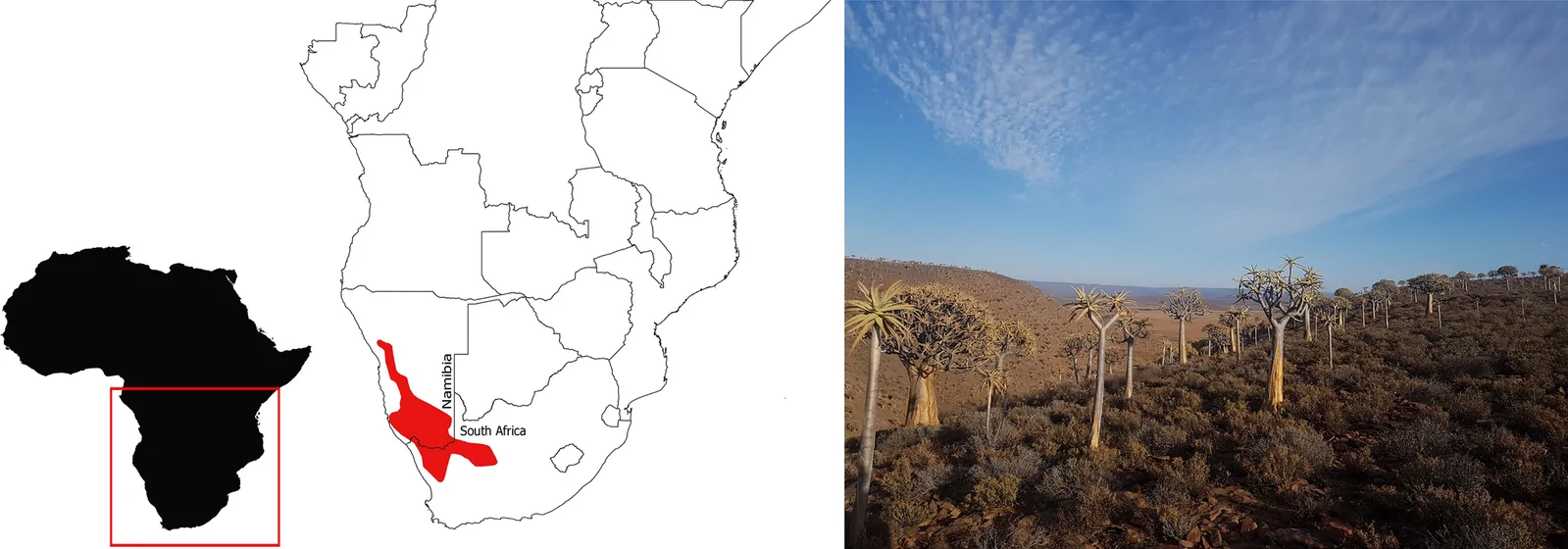
Geographical range of *Aloidendron dichotomum* and picture. Photo credit Kerry-Anne Grey

*Aloidendron dichotomum* is an iconic, endemic tree-aloe of southern Africa (Grace et al. 2009). Its current distribution covers a large area from the Brandberg Massif in the arid central plateau of Namibia to its southerly limits in South Africa’s Namaqualand and Bushmanland (Van Wyk and Smith 2014; Guo et al. 2016) (Fig. 2).

#### Climate change impacts

In the past few decades, substantial dieback in the warmer climate populations of *A. dichotomum* has occurred (Foden 2002; Foden et al. 2007; Van der Merwe and Geldenhuys 2017) with increased productivity and recruitment in cooler, typically poleward, populations (Foden et al. 2007; Van der Merwe and Geldenhuys 2017). This pattern is consistent with a climate-induced range shift produced by the current rate of warming in this region (MacKellar et al. 2014).

#### Nature for nature (NN)

**Fig. 3:**
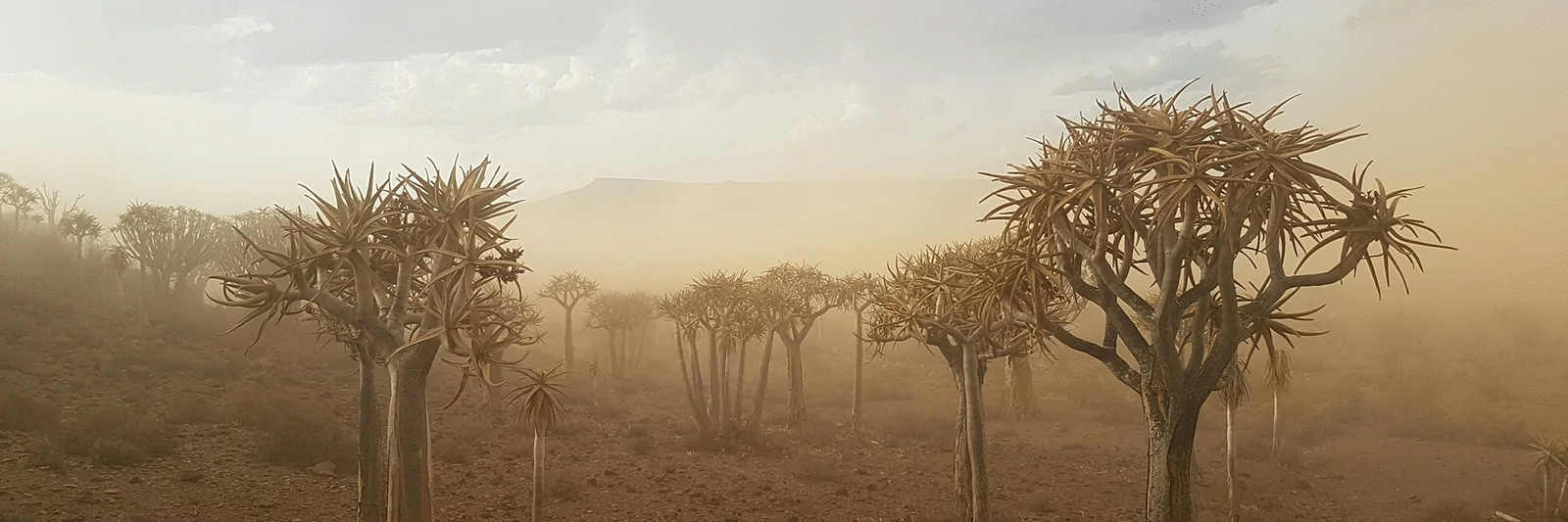
*A. dichotomum* is used by traditional San healers in both Namibia and South Africa for medicinal purposes (NC value). Photo credit Kerry-Anne Grey

*Aloidendron dichotomum* grows to 10 m tall in scattered to dense stands (sometimes referred to as “forests”), in areas that rarely support tree life (Fig. 3) (Foden 2002). These trees have been present in the arid regions of south-western Africa for over 300 years, and form a vital component of the habitat, with *A. dichotomum* identified as a keystone species in these areas (Midgley et al. 1997; Van der Merwe and Geldenhuys 2017). As climate change impacts the historic habitat of *A. dichotomum*, the decline of these populations would present a significant loss with consequential impacts on the species assemblages that aggregate around them.

#### Nature for society (NS)

It is extremely unusual to find forest-like stands in deserts. As a consequence, *A. dichotomum* populations are key for local people as they attract tourists, providing economic value for the ecotourism industry. Climate change-related shifts in the species' distribution and aesthetic changes in the forests lower their potential to attract tourists (*Pers. comm. with local stakeholders*). As a result, not only does the loss of this species from the landscape pose a threat to local revenue, but drought-related impacts may also adversely affect tourism in these areas.

#### Nature as culture (NC)

*Aloidendron dichotomum* (known as quiver tree, kokerboom, or Choje in South Africa, Namibia, and indigenous communities, respectively) is a cultural emblem of South Africa and Namibia. Centuries ago, the San hunters would hollow out its branches to use as quivers for their arrows, hence the name quiver tree (Foden 2002) and healers would use this species for medicinal purposes to treat respiratory conditions such as asthma and diseases such as tuberculosis (Grace et al. 2009). Moreover, *A. dichotomum* is recognized as the national tree of Namibia featuring on the country’s 50 cent coins emphasizing its cultural importance (Foden and Midgley 2009). Loss of such a culturally important plant would have negative repercussions for these communities where it represents a fundamental part of their cultural identity.

#### Current management options and the NFF

There is no coherent conservation strategy being implemented to address the risk of climate change for *A. dichotomum* but rather a “wait and see” response with the establishment of formal monitoring protocols across as many populations as possible (Van der Merwe and Geldenhuys 2017). However, there are a number of nurseries that currently have the ability and expertise for target protection of vulnerable populations by establishing seedlings, with the potential for assisted translocation responses and ex situ conservation (Table 2).

**Table 2:**
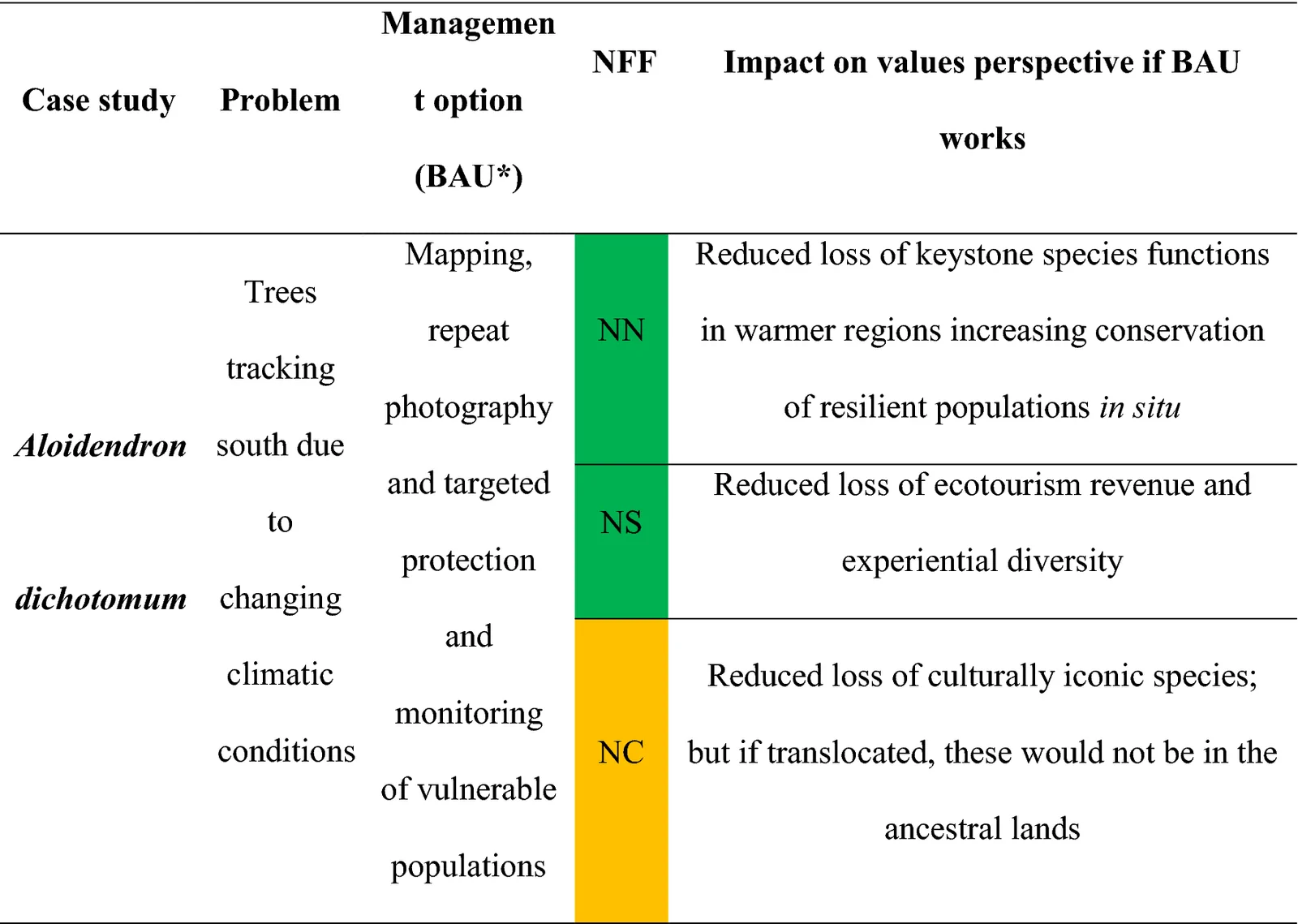
The *Aloidendron dichotomum* case study and how its management option (business as usual—BAU) in the face of climate change unfold under different NFF value perspectives

Color code: green for overall positive feedback; orange, trade-offs; red, negatively impacted

### Woody encroachment in savannas

**Fig. 4:**
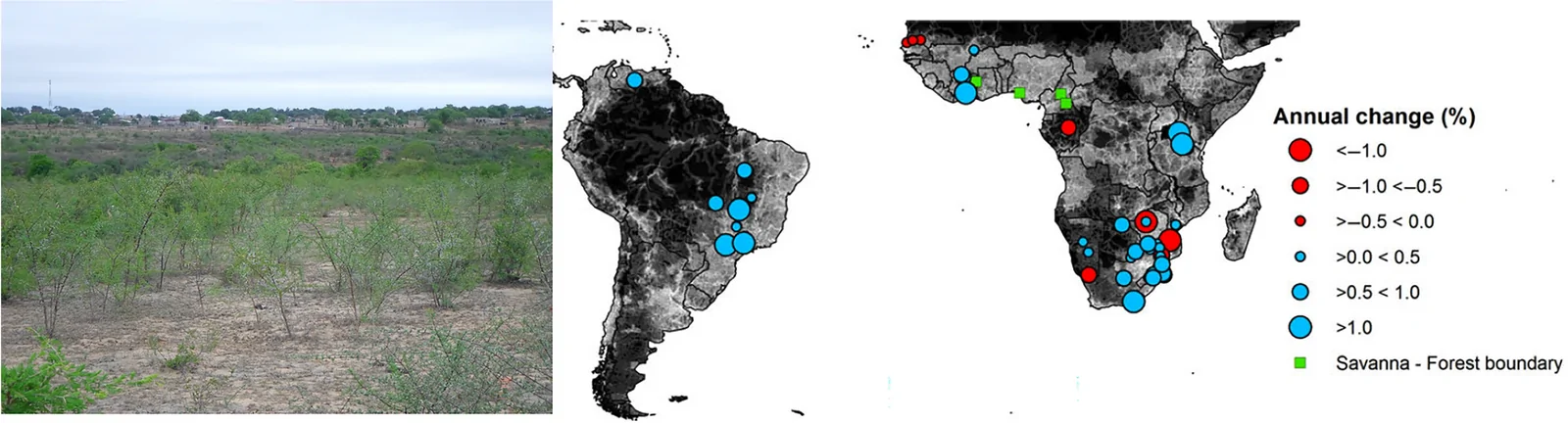
Woody encroachment, and regions with reported changes in woody cover in grassy ecosystems of South America and Africa. Photo credit Izak P. J. Smit. Map adapted from Stevens et al. (2017)

Savannas can be defined as a mosaic of dynamic systems with varying spatial–temporal ratios of open and closed patches of grass and tree cover. These ratios of patches are maintained by a combination of bottom-up processes (i.e., soils and climate) and top-down disturbances (e.g., fire and herbivory) (Sankaran et al. 2005). Processes that homogeneously and consistently favor one growth form over the other across large scales are a threat to savanna ecosystems (Fig. 4). This is the case of woody encroachment, a process where a hard-to-reverse increase in woody cover decreases herbaceous biomass at multiple scales (Smit et al. 2016).

#### Climate change impacts

Increased atmospheric carbon dioxide (Donohue et al. 2013) and changes in rainfall regimes are often considered the most likely causes for woody encroachment in areas where land use and management practices have stayed stable (Stevens et al. 2017; Rosan et al. 2019; Buitenwerf et al. 2012). Despite large regional variation, savannas in southern Africa and South America have seen a 2.5% and > 7% increase in average woody cover encroachment per decade since the 1970s, respectively (Stevens et al. 2017). It is worth noting that the implications of climate change to the process may not be unidirectional as they could be both beneficial and detrimental to biodiversity (Meik et al. 2002; Smit and Prins 2015; Ratajczak et al. 2012).

#### Nature for nature (NN)

**Fig. 5:**
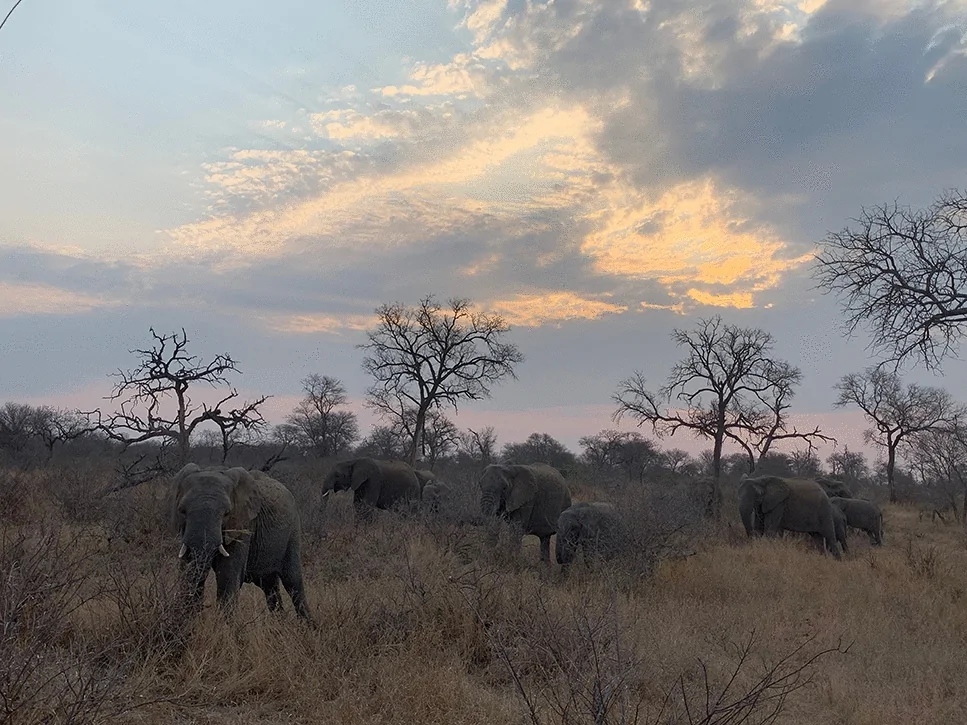
Elephants in the Greater Kruger National Park playing their role in limiting woody encroachment (NN). Photo credit Laura Pereira

High or continuous woody cover can influence individual species and/or taxonomic groups (Meik et al. 2002; Smit and Prins 2015) as well as biological diversity more broadly (Ratajczak et al. 2012). However, it does not always lead to degraded ecosystems (Eldridge et al. 2011). Increased woody cover favors large browsing mammals due to increased forage availability, but results in lower grazer densities due to the associated reduction in herbaceous biomass (Fig. 5) (Smit and Prins 2015). In addition, the consequent reduction of herbaceous biomass can shift fire regimes producing fewer and lower intensity fires (Smit and Prins 2015).

#### Nature for society (NS)

The substitution of woody encroachment for grazing grass may have large implications for subsistence grazing systems as in many African societies, where cattle are incredibly important (Dalle et al. 2006; Shackleton et al. 2005; Luvuno et al. 2018). Moreover, tourists indicate that they would avoid woody encroached savannas where game viewing may be less rewarding (Gray and Bond 2013). However, increased woody cover can also have positive implications for humans, especially in rural and/or poor areas where the wood can be used as a source of energy for cooking and heating (Madubansi and Shackleton 2007).

#### Nature as culture (NC)

An increase of woody encroached areas may influence the lifestyle, culture and identity of rural communities. In some African societies, cattle are incredibly important as symbols of wealth and power, and are utilized for a range of cultural purposes such as payment for fines and brides (“*lobola*”) (Shackleton et al. 2005; Luvuno et al. 2018). Moreover, firewood is very much part of the African culture and socializing next to wood fires is a common social practice. There also seems to be a preference in some societies for tourism in open savanna landscapes with scattered trees and an open and grassy understory (Sharp et al. 2012).

#### Current management options and the NFF

Various localized management interventions are implemented to reduce woody cover encroachment. These include mechanical clearing, poisoning of woody plants (sometimes through aerial spraying) (Bezuidenhout et al. 2015) or changed fire regimes (increased fire intensity and frequency) (Smit et al. 2016). Even though these interventions may sometimes be successful at a local scale in reducing woody cover, there are trade-offs, and they may have to be continuously repeated since the driver of change (i.e., increased carbon dioxide) is not addressed (Table 3).

**Table 3:**
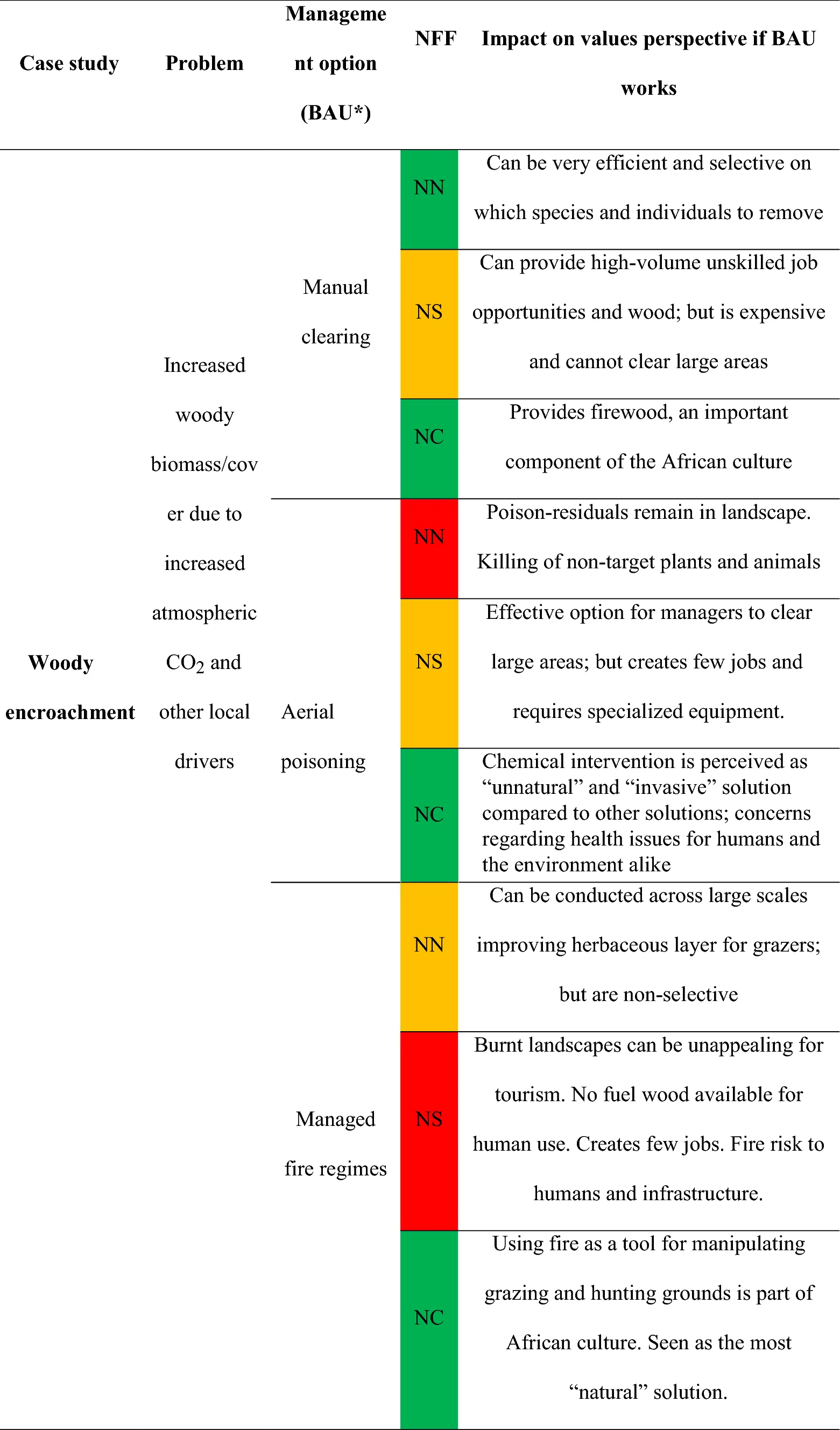
The woody encroachment case study and how the different management options (business as usual—BAU) in the face of climate change unfold under different NFF value perspectives

Color code: green for overall positive feedback; orange, trade-offs; red, negatively impacted

### The Galapagos Marine Reserve

**Fig. 6:**
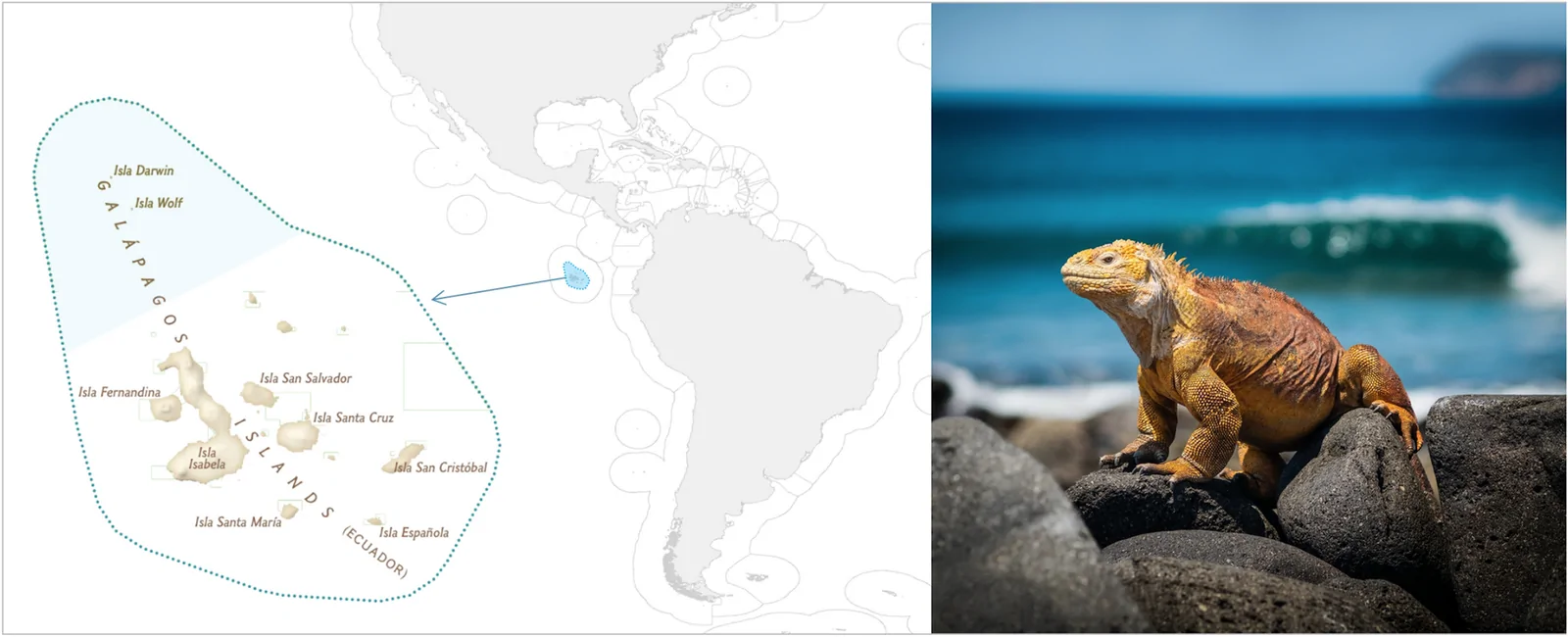
Location of the Galapagos Archipelago in the Pacific ocean, and limit of the Galapagos Marine Reserve (map adapted from the Galapagos Conservation Trust). Photo credit Simon Matzinger

The Galapagos Archipelago, located in the eastern Pacific Ocean, 1,000 km off the coast of mainland Ecuador, includes twelve islands that emerged from the sea millions of years ago (Barragán-Paladines and Chuenpagdee 2015). In 1998, the Galapagos Marine Reserve (GMR), a multi-use marine protected area, was created around the Galapagos Islands (Moity 2018; Barragán-Paladines and Chuenpagdee 2015) (Fig. 6). The region is a hotspot for biodiversity with a high level of endemicity and biomass of pelagic species (Edgar et al. 2008; Barragán-Paladines and Chuenpagdee 2017).

#### Climate change impacts

Rising ocean temperatures, ocean acidification, and increased frequency and intensity of extreme weather events (such as El Niño) are among the factors that make the Galapagos extremely vulnerable to the impacts of a changing climate (Sachs and Ladd 2010). In the last 30 years, El Niño events have been less frequent but more intense when compared to records from the past 400 years (Freund et al. 2019), having significant impacts in the GMR social-ecological systems (Salazar and Denkinger 2010; Boersma 1998; Vargas et al. 2007). Moreover, the frequency and intensity of extreme weather events is expected to increase in the upcoming decades (Frölicher et al. 2018; IPCC 2019).

#### Nature for nature (NN)

The GMR provides habitat to more than 2900 marine species from a variety of groups from invertebrates to large marine mammals (Edgar et al. 2008; Barragán-Paladines and Chuenpagdee 2017) with over 18% being endemic to the region (Moity 2018; Markham et al. 2016). This high level of endemism also extends to terrestrial species (Markham et al. 2016), making the Galapagos a biodiversity hotspot. As climate-related drivers of change may induce the redistribution of marine life (Pecl et al. 2017), Galapagos endemic species and overall biodiversity might be increasingly at risk (Salinas-de-León et al. 2020; Salazar and Denkinger 2010).

#### Nature for society (NS)

**Fig. 7:**
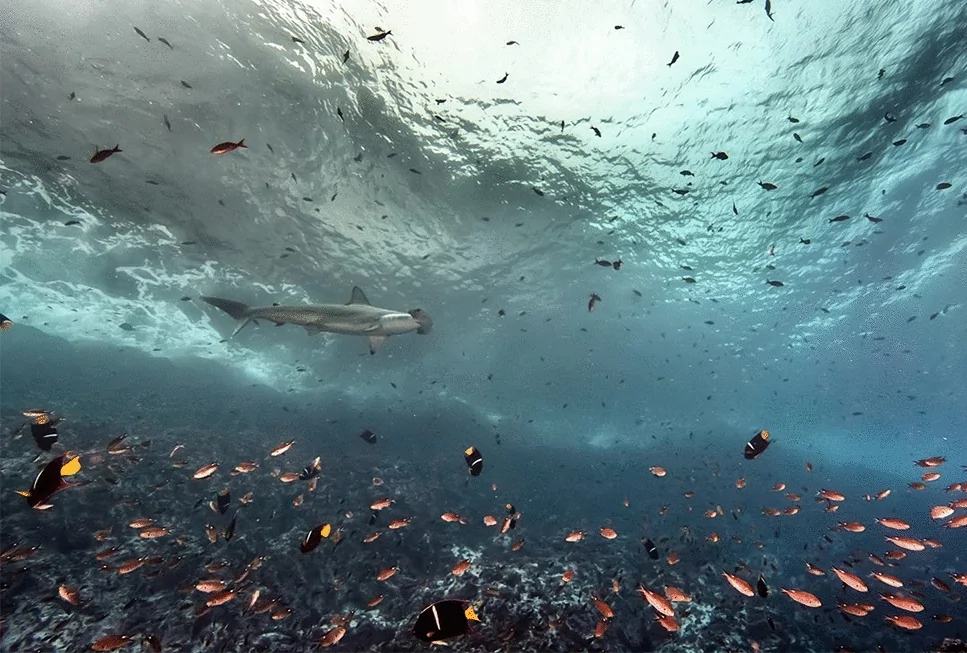
Marine-based tourism is the main source of income for many *Galapagueños*. Photo Credit Juan Sebastián Mayorga

Tourism is the primary driver of economic development in the archipelago, and is growing exponentially (Walsh and Mena 2016). Tourism management in the GMR seeks to balance the high demand of the sector with the pristine habitats that tourists desire (Markham et al. 2016) (Fig. 7). Another relevant human activity pertains to fisheries, mostly composed of small-scale industries (Barragán-Paladines and Chuenpagdee 2017; Markham et al. 2016). There has been substantial political dynamics around the Galapagos marine reserve between the two groups (Barragán-Paladines and Chuenpagdee 2017). Climate-induced changes in the distribution of fish stocks and charismatic marine species (e.g., whales) together with more intense and frequent storms are expected to substantially affect both fisheries and tourism (Santos et al. 2016). Changes to either tourism or fishing would detrimentally effect livelihoods in the region, regardless of the social-ecological dynamics.

#### Nature as culture (NC)

Unlike other archipelagos with long histories of human settlement, there is no native/indigenous population in the Galapagos (Walsh and Mena 2016). Therefore, the somewhat recent “*Galapagueño*” identity pertains to people that inhabit the Galapagos islands, who are originally from other places, and that are connected by living in close connection to a vulnerable and unique ecosystem (Barragán-Paladines and Chuenpagdee 2017; Salinas-de-León et al. 2020). The Galapagos uniqueness regarding its marine biodiversity has led to the recognition of the archipelago as a UNESCO Natural Heritage Site for Humanity in 1978, and the classification of the GMR itself as a UNESCO Natural World Heritage Site in 2001 (Moity 2018).

#### Current management options and the NFF

Monitoring of climate change in the Galapagos Islands is extensive (GCT 2019), with international teams of scientists measuring and tracking weather patterns and animal behavior (Manzello et al. 2014; Sachs and Ladd 2010; Salazar and Denkinger 2010). Conceptually, it is well recognized that improving the understanding on impacts of climate change on the GMR social-ecological systems is of paramount importance to informing future conservation management (Moity 2018; GCT 2019). Nevertheless, despite the GMR being a protected area with the potential of supporting communities facing climate change (Davies et al. 2017), specific actions to respond to these challenges are still to be implemented (Table 4).

**Table 4:**
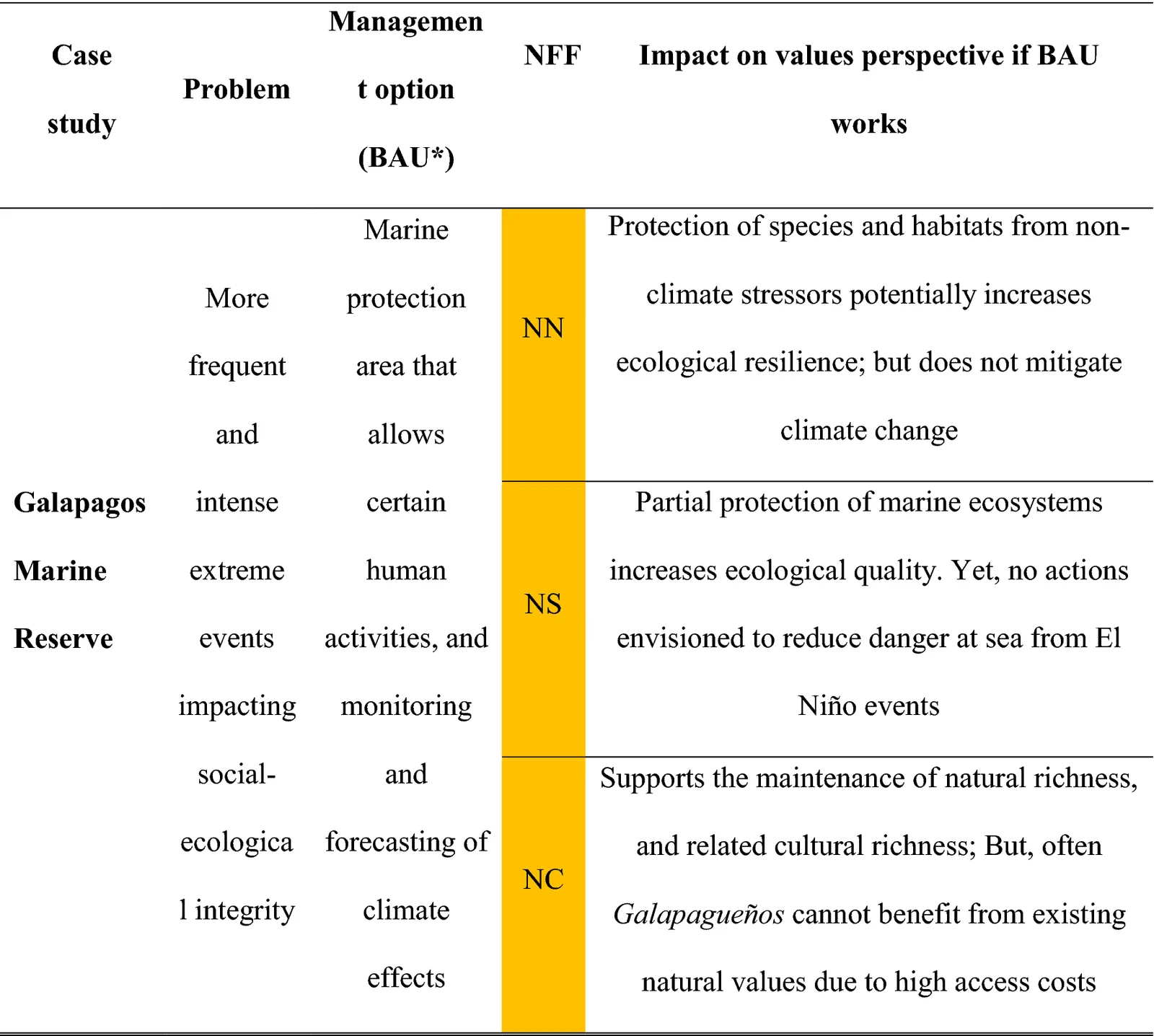
The Galapagos Marine Reserve case study and how the different management options (business as usual—BAU) in the face of climate change unfold under different NFF value perspectives

Color code: green for overall positive feedback; orange, trade-offs; red, negatively impacted

## Discussion

In this paper, a group of scientists and practitioners from different disciplines and backgrounds used three case studies (Table 1) to discuss a recently developed framework that recognizes multiple futures for nature and society. In this section, we discuss the NFF as a heuristic tool effective in surfacing implications under different management strategies related to one of the NN, NS and NC value perspectives, and where there are gaps in knowledge to fully understand the impacts of different management decisions.

### Cross-case study comparison

#### Nature for nature (NN)

According to an ecocentric perspective, nature is valuable in and of itself without needing to benefit humans directly and thus there is a value in the conservation of nature’s diversity and ecological functions (Piccolo et al. 2018). Targeted protection against climate change for *A. dichotomum* and manual clearing of woody encroachment are the only current management responses that address a NN perspective. Monitoring and mapping of threatened populations to support assisted translocation of *A. dichotomum* reduces the loss of such keystone species, while manual clearing of woody encroachment provides a target solution with minimum disturbance to the system (Foden et al. 2007). On the other hand, aerial poisoning on savanna systems was identified as the only management option negatively impacting nature for its potential to remove non-target species (Bezuidenhout et al. 2015). In some cases, benefits will be context dependent within the NN component. For woody encroachment, the benefits of fire as a natural disturbance factor are crucial in fire-prone savanna systems. However, there is often some ecological cost in terms of non-target species (e.g., loss of tall trees and coppicing of encroacher species). Moreover, there are policy constraints for burning high intensity fires due to the risks of wildfires, an important concern as these become more intense and harder to control under climate change. Similarly, while the implementation of the multiple-use MPA in the Galapagos does not directly address climate change impacts from stronger El Niño events, it does contribute to increasing the overall ecological resilience of the ecosystem, by protecting species and habitats from local human pressures such as overfishing, marine pollution, or habitat destruction.

#### Nature for society (NS)

Nature provides important benefits to society, providing protection from extreme events, functioning as a main source of food, or by sustaining livelihoods. Ecotourism, for example, was identified as an important contribution across the three case studies. For both *A. dichotomum* and the GMR, the current management options will potentially reduce losses to ecotourism, however, burned savanna landscapes for managing woody encroachment can be unappealing, and thus less of an attraction to tourists. As an alternative, increased woody encroachment could be addressed by manual clearing, creating unskilled jobs and providing wood for households. However, from a landowner’s perspective, this is less efficient and more costly than mechanical clearing that creates fewer jobs but covers larger areas. Similarly, the GMR balances high tourism demands with pristine ecosystems that tourists look for, but limits the fishing grounds of local fishers within the GMR (Barragán-Paladines and Chuenpagdee 2017; Walsh and Mena 2016). Although management interventions need to negotiate these different values (e.g., tourism and fishing), both will be negatively impacted by climate change events such as more frequent and intense El Niño events.

#### Nature as culture (NC)

Human societies can be perceived as an integral part of nature and its functions, rather than just resource consumers (Chan et al. 2018). In the current management options, only manual clearing and appropriate fire regimes for controlling climate driven woody encroachment are positively related to cultural values as firewood, from manual clearing, is very much part of African culture for cooking, heating and socializing, while appropriate fire regimes is a tool used over generations for manipulating grazing and hunting grounds (Luvuno et al. 2018). This is not the case of *A. dichotomum* and the GMR case studies. Monitoring and implementing assisted translocation of the species *A. dichotomum* might reduce the loss of a culturally iconic species; however, it does not address the species shifting further away from local communities (e.g., the San healers who are culturally as well as physically dependent on the tree). While Western medicine may be able to substitute for the medicinal uses of the plant (albeit is not the same), the loss of a deep cultural connection to the tree as it is embedded in place, folklore and culture cannot be substituted in the same way. A similar pattern is seen in Galapagos, where the GMR increases the system’s ecological resilience but where the impacts of climate change may irrevocably alter the ecosystem. Here, the NC benefit will be dependent on the sectoral interests. On the one hand, the global population puts a high cultural value on preserving the World Heritage Site. However, many *Galapagueños* cannot benefit from the unique local marine ecosystems because of the high cost of tourism that limits the access to protected places (Barragán-Paladines and Chuenpagdee 2017).

### Making the trade-offs explicit and identifying co-benefits

As is clear above, the NFF provides a way to make trade-offs between values and management options explicit, allowing decision-makers to have a broad picture of the social-ecological system including the effects of certain policies on different contributions of nature to people. Such trade-offs between different impacts can even occur within one value perspective, as exemplified by the woody encroachment case study. An increase in woody encroachment in savannas will negatively impact NS value for farmers as it reduces space for cattle grazing, potentially impacting commercial and subsistence grazing systems (Dalle et al. 2006). Addressing this issue by manually clearing the area can benefit the poor by providing high-volume unskilled job opportunities and household wood. However, it is not very efficient as it cannot cover very large areas. Thus, a farmer would likely prefer to use mechanical clearing that requires less employees and can cover larger areas. In both cases, the effects of the strategy from an NS perspective can be positive or negative, and, therefore, another layer of analysis that understands the context and diversity of stakeholders is key in the implementation of policies.

Trade-offs could also appear between and within values simultaneously, as seen in the case of the Galapagos. Here, different social and economic values (NS) need to be considered when making decisions on the use of marine resources, as different human uses of the ocean space (in this case, fisheries, tourism and conservation) compete for the same area (Heylings and Bravo 2007). Establishing a marine conservation area (the GMR) can be positive from a variety of perspectives, such as the NS perspective from the ecotourism industry that requires healthy ecosystems to take place, or the NN and NC perspectives from the international community that recognize the islands as a biodiversity hotspot and a heritage site for humanity. However, having a protected area in place will negatively affect the NS perspective of local fishers that find their fishing area reduced (even when there is fish spillover from the MPA that partially compensates fishers, Halpern et al. 2010), or the NC perspective from local inhabitants that cannot afford to visit the GMR (Barragán-Paladines and Chuenpagdee 2017).

In thinking about climate change adaptation pathways, it is important to recognize that there are trade-offs and multiple options that are additional to the current trajectory (Fazey et al. 2018). By having alternative responses available, it is possible to illustrate these diverse pathways and not only the dominant ‘highway’ or the ‘Business as Usual’ (BAU) approach (Leach et al. 2010). Examples of BAU or dominant approaches in conservation include the establishment of conservation areas that exclude people from accessing resources on which they were previously reliant (Chirico et al. 2017; Kubo and Supriyanto 2010). In the long term, these approaches may fail or result in undesirable outcomes, such as “illegal” resource extraction (e.g., when fishing grounds that once were open suddenly become illegal) (e.g., Chirico et al. 2017; Kubo and Supriyanto 2010). However, when diverse viewpoints are taken into consideration, different, more inclusive options may emerge such as community-based natural resource management (e.g., Chirico et al. 2017; de la Lama et al. 2018; Palacios-Abrantes et al. 2018), although this can also be contentious (Chaigneau and Brown 2016). While we specifically focused on trade-offs, it is worth mentioning that the NFF could also be used to find synergies between values and management options.

### What we put into models influences the outcome for management

Often decision-makers and managers rely on model outputs for understanding the potential futures that might unfold and, therefore, the kinds of solutions that need to be implemented (e.g., Gattuso et al. 2015; Obermeister 2017). Thus, finding ways in which the different components of the NFF can be included could help modeling efforts represent a broader set of societal values that encapsulate a wider range of nature’s contributions to people. What we put into models is determined by the characteristics underlying their initial scenario assumptions (O’Neill et al. 2014) and so we need to start with broader ideas of diverse values when looking at scenarios through a critical social science lens (PBL 2018; Rosa et al. 2017). For example, a model framework for managing woody encroachment only considering NS values could result in moving away from using fire as the potential for large-uncontrolled fires increases under more frequent extreme weather events (IPCC 2021). However, by not considering NC and NN perspectives, the model is ignoring that using fire as a tool is an important part of African culture and often seen as the most “natural” solution, with appropriate fire regimes important in fire-adapted ecosystems. This example highlights how prioritizing different value perspectives will have implications for how climate change impacts are described, and further will make explicit what management options are being prioritized (O’Neill and Nakicenovic 2008).

Using the NFF triangle to demonstrate the need for a range of options based on a plurality of value perspectives also brings up issues of resource constraints and the need to make more complex conservation decisions. Importantly, climate-ready strategies that do not consider, or indirectly foster, inequality can have negative outcomes making socially or culturally marginalized communities especially vulnerable to climate change (Adger et al. 2013). It is, therefore, imperative that biophysical models are better able to represent these diverse values in their models in order to help make more informed decisions for social-ecological systems under climate change (PBL 2019; Chan et al. 2018; Pascual et al. 2017; Piccolo et al. 2018). Opening up diverse options and management pathways requires better analysis of the different alternatives and, therefore, new indicators that can be fed to new, improved models (Siqueira-Gay et al. 2020). Current indicators still refer to an inherently high species biodiversity value, emphasizing the NN perspective, but potentially leaving out areas with a high NS or NC value. In many instances, it will be difficult to develop indicators (e.g., to quantify cultural threats under climate change) that talk to these more diverse values, especially in the short term, but it is important to try and find alternative ways. The need for developing indicators that reflect the full range of the NFF triangle is imperative if the framework is to be operationalized. Merely defining new indicators is, however, insufficient as it will be necessary to define baselines and targets to be managed towards.

The need to be able to model more diverse values will, therefore, become increasingly important as more organizations and schemes recognize that single indicators are insufficient. For example, instead of only modeling for commercial fish biomass under climate change, the impacts on specific iconic or culturally important species could also be explicitly recognized as well as the knowledge from the communities culturally bonded to these species (Vierros et al. 2020). Recognizing multiple values for nature has implications for management decisions under climate change, where both trade-offs and co-benefits can be highlighted, and provides an opportunity for decolonializing biodiversity conservation praxis (Lyons et al. 2020). As argued by Van Kerkhoff et al. (2019), there is a need to shift from seeing climate adaptation as a purely scientific issue to understanding it as a governance issue embedded within a politics of place and history. Thus, incorporating ecological knowledge and indigenous communities’ values in models becomes essential for developing successful management plans in a changing world (Vierros et al. 2020). Lessons can be learned from different communities on how they perceive and adapt to climate change as cultural heritage such as indigenous knowledge and a holistic view of community and environment are major resources for adapting to climate change (ICOMOS 2019; IPCC 2014a, b).

### Benefits and challenges for future research and management

Employing the NFF more clearly in a variety of case studies can have an important role for opening up discussions across knowledge systems on how to manage species and ecosystems more effectively. In addition, the NFF approach has direct implications for the management of conservation areas. Allowing for multiple values means that management of such sites does not necessarily need to conform to historical global ideas of conservation under which many of these areas were originally established, which typically referenced NN values (Hingston 1931). Instead, a focus on more inclusive ‘new conservation’ (Kareiva and Marvier 2012) is encouraged. On the flip side, the NFF allows managers to keep the utilitarian approach to nature in check too, i.e., an area, system, or species does not only have a use for the ecosystem services it provides, but can also be managed for its intrinsic or cultural value. If, for example, we needed to restore a degraded water catchment system, an utilitarian approach would plant water retaining species even if it reduced the biodiversity or cultural significance of an environment, but acknowledging NN and NC value perspectives of a diverse catchment ecosystem might generate alternative options such as rehabilitating a wetland, which might be more financially costly, but would meet more goals in the long term (Nelson et al. 2009).

However, having diverse pathways does not necessarily lead to more straightforward decision-making. It could be challenging for managers to make a decision considering the trade-offs underlying different indicators within a value (e.g., trade-offs between job opportunities for fishing versus tourism industry under NS), as well as the trade-offs between multiple dimensions (e.g., NC, NS, and NN) rather than just one. In some instances, such trade-offs combined with governance characteristics (e.g., influence of power in decision-making) may become so challenging that no decision is made (Barragán-Paladines and Chuenpagdee 2015). Yet, developing the science that can make these values explicit and then offer management options that address each of these is critical (Barragán-Paladines and Chuenpagdee 2015), especially given the threat of climate change that will impact how these nature values manifest in the future. The NFF enables this transparency in the decision-making process, but it also highlights limitations to the application of the NFF given resource constraints and power dynamics in the decision-making process. What also remains challenging when using the NFF is that trade-offs often require very different indicators of success (e.g., number of jobs created vs number of bushes removed), which can be challenging to measure and implement from a modeling and decision-making perspective.

## Conclusion

In this overview, we aimed to identify the potential use of the NFF as a tool for recognizing multiple (desired) futures for nature under climate change. Our findings show that by considering multiple values of nature, adaptation plans could be better suited to face the social-ecological consequences of climate change, while providing an array of options beyond the BAU approach that could lead to more equitable outcomes for nature’s contribution to people. Following a holistic approach, where policies and management decisions are made addressing multiple values of nature, can support effective policy towards future-oriented conservation under climate change. Moreover, the NFF allows for the identification of trade-offs between values and within stakeholders of different policies. A more holistic appreciation of the trade-offs and co-benefits involved in conservation management decisions is possible by making diverse values for nature more explicit. While the NFF provides a mechanism for policymakers to make more informed decisions on biodiversity management, its operationalisation will require an unprecedented degree of engagement between stakeholders, policymakers, natural and social scientists to properly account and differentiate between values as well as incorporate qualitative metrics into modeling frameworks. This study constitutes a first attempt to apply the NFF based on experts’ opinions to cases where climate change is impacting social-ecological systems requiring the development of a diverse set of pathways. Further studies should focus on empirically testing the NFF in real-life situations as climate change continues to impact social-ecological systems globally.
